# Leadership career expectations of physicians and dentists in specialist education

**DOI:** 10.1108/LHS-10-2025-0164

**Published:** 2026-07-27

**Authors:** Tiina A. Tuononen, Annariitta Kottonen, Rosa Korhonen, Anna Liisa Suominen, Pia Heilmann

**Affiliations:** Faculty of Health Sciences, Institute of Dentistry, University of Eastern Finland, Kuopio, Finland; Independent Actor, Kauniainen, Finland; Faculty of Health Sciences, Institute of Dentistry, University of Eastern Finland, Kuopio, Finland, and Odontology Education Unit, Kuopio University Hospital, Kuopio, Finland; Faculty of Social Sciences and Business Studies, Business School, University of Eastern Finland, Kuopio, Finland

**Keywords:** Leaders, Doctors, Education, Healthcare, Management

## Abstract

**Purpose:**

The purpose of this study is to examine specializing physicians and dentists’ perceptions of management and leadership, their interest in managerial work and the role of management in their future careers. Studying this topic is important because many of these specialists will become future leaders in healthcare.

**Design/methodology/approach:**

The target group of the study consisted of 114 physicians and dentists attending a multidisciplinary management training course from 2023 to 2024 during their specialist training. The data were collected using a Webropol questionnaire, including structured and open-ended questions, and analyzed with quantitative and qualitative methods. The response rate was 32.5%.

**Findings:**

Respondents’ views on management roles included perceptions of the roles of leader, resource allocator and entrepreneur. Three-quarters of the respondents believed that management and leadership would be part of their future careers. Their thoughts and assumptions regarding their future roles as specialized physicians or dentists suggest that, ten years from now, the role of expertise will become significantly stronger. However, they also anticipated that the roles of management and leadership would become more prominent in the future.

**Originality/value:**

It is important to understand specialized physicians’ and dentists’ expectations about management and its role in their future to be able to prepare these professionals for their future responsibilities. Whether they want it or not, management will be a part of their work as specially trained professionals. Most of them will lead teams as operational-level managers, while some will advance to higher management positions.

## Background

### Healthcare management challenges

Healthcare leaders play crucial roles in overseeing daily operations within their organizations. Their responsibilities encompass a wide range of tasks, including human resource management, financial oversight and developmental initiatives. Their role is especially demanding during reforms and structural change implementation, which have been very common in many countries in recent decades (Aggarwal *et al.*, 2023; Degeling and Carr, 2004; Dong *et al.*, 2014; Ravaghi *et al.*, 2018). In Finland, social and healthcare services are undergoing major changes due to financial sustainability, growing demand for services and staff shortages (Ministry of Social Affairs and Health, 2025).

The number of people working in health and social services in Finland has been continuously growing and is now approximately 438,000 (Statistics Finland, 2025). However, there is still a significant labor shortage in the healthcare and social welfare sectors (Tuomaala, 2024). An additional challenge in healthcare leadership is multiprofessionalism. Healthcare services are increasingly often produced by professionals working in multiprofessional teams (van Diggele *et al.*, 2020; Parviainen *et al.*, 2018). Management and leadership skills, along with an interest in leadership, are necessary when working in the current challenging healthcare operating environment.

### Physicians and dentists as health care leaders

In Finland, the leadership role of physicians is laid down in the Health Care Act (Health Care Act 1326/2010, 2025). According to the Act, each healthcare unit must have a physician-in-charge who is responsible for the health and medical care provided at the unit. The physician-in-charge shall oversee all provisions of health and medical care in the unit. In addition, the policy of the Finnish Medical Association (2014) states that any physician primarily engaged in clinical work must be supervised, both administratively and professionally, by another physician. In a study on the careers of healthcare managers, respondents with a physician background emphasized the importance of the unit leader’s familiarity with the clinic’s operations, the staff they manage and the patients. They viewed the trend of appointing managers from outside the healthcare sector as undesirable (Vistbacka, 2019, p. 67). There is some evidence suggesting that physicians in leadership roles may promote improved patient care outcomes and more effective healthcare organization performance compared to nonmedical leaders. However, the evidence remains inconclusive (Falcone and Santiani, 2008; Goodall, 2011; Mohmad *et al.*, 2024; Onyura *et al.*, 2019; Sarto and Veronesi, 2016; Tasi *et al.*, 2017). During undergraduate training, physicians and dentists acquire competencies that can also be valuable in managerial or leadership roles. For example, physicians and dentists possess a good understanding of healthcare systems and patient care (Angood *et al.*, 2014), knowledge that professionals from other fields, such as engineering or economics, may not inherently have.

Physicians and dentists often end up in management and leadership positions based on their academic merits, not necessarily thanks to their management skills or background (Ham *et al.*, 2011; Quinn and Perelli, 2016). It is also often assumed that good clinical expertise would compensate for deficiencies in leadership skills (Huikko-Tarvainen *et al.*, 2021; Lüchinger *et al.*, 2024; Satiani *et al.*, 2014). Physicians may not inherently possess all the competencies required for effective leadership. Therefore, targeted training is essential to help them develop the necessary skills for managerial and leadership roles. (Silbaugh and Leider, 2009).

### Management and leadership as an essential part of the work of medical and dental experts

Expert jobs and tasks are increasingly hybrid, requiring a combination of different skills and talents (Jännäri *et al.*, 2018). According to Eriksson (2006), an expert is a person who is a widely recognized source of knowledge, skill or technique and whose judgment and status are recognized publicly or by peers. Expertise includes nine key components: competence, skills, teaching, self-leadership, learning, networking, innovation, problem-solving and understanding the big picture. Expert work requires formal training, qualifications and work experience. In an expert organization, expertise and leadership are intertwined (Heilmann, 2022).

The rigorous education and training that physicians and dentists undergo prepare them to be expert clinicians rather than experts in management, and individual problem-solvers rather than collaborative leaders (Guevara *et al.*, 2020; Rotenstein *et al.*, 2018). Nevertheless, all physicians and dentists have clinical leadership roles, which are a part of their clinical work in a healthcare team (Berghout *et al.*, 2017; Blumenthal *et al.*, 2012).

Many physician and dentist leaders seem to play a hybrid role by practicing medicine and serving in a leadership role at the same time. As this dual role may place them in a position of conflicting priorities, some have questioned the effectiveness of this model (Kippist and Fitzgerald, 2009). Physician leaders’ work comprises both general management/leadership activities and medicine. Medical leadership is defined to include the tasks that physicians, either in formal managerial roles or acting as informal leaders, perform in their daily leadership work to influence others toward goal attainment (Berghout *et al.*, 2017). In the healthcare business, understanding how to practice medicine is important. However, healthcare leaders also need to have multidimensional competency and an understanding of cross-cutting influences (Day and Harrison, 2007).

Specialization training enables physicians and dentists to attain enhanced expertise within their specialty. However, despite this strong clinical expertise, management and leadership tasks will still be part of their professional role, and progressing in the career often either means or will lead to taking on a supervisor position – whether willingly or reluctantly. As a result, it is essential to incorporate management and leadership objectives into specialization training to better prepare professionals for their evolving responsibilities.

## Aim

The aim of this study was to examine specializing physicians and dentists’ perceptions of management and leadership, their interest in managerial work and the role of management in their future careers.

## Materials and methods

### Study background

In 2023, there were approximately 23,300 working-aged physicians in Finland, of whom 71% had completed specialist training (Finnish Medical Association, 2025). Accordingly, there are about 4,500 working-aged dentists, of whom 15% have trained as specialists (Finnish Dental Association, 2025). In Finland, an extensive reform of specialist training is currently underway, aiming to enhance the quality of education and ensure an adequate number of specialists across the country. The coordination division for specialist medical and dental training, operating under the Ministry of Social Affairs and Health, has drawn up action plans for the periods 2017–2019 and 2023–2027, according to which the training has been developed to become more competence-based medical and dental specialist education (Ministry of Social Affairs and Health, 2023).

Since 2009, the training of specializing physicians and dentists in Finland has included mandatory management and leadership studies amounting to ten European Credit Transfer and Accumulation System points. These studies can be completed at any stage of the training. The nationally defined study content includes human resource management, interaction and communication; the structure, operation and law of social and health care; the health and social services system; and health care funding. The aim of the training is to provide the required theoretical background knowledge needed for team leader positions in the field of medicine (Lääketieteelliset.fi, 2025).

### Data collection

The target group consisted of 114 physicians and dentists undergoing their specialist education. They were participating in multidisciplinary leadership and management studies in the period 2023–2024 at the University of Eastern Finland. The number of respondents was 39, of which 37 (32.5%) gave research permission.

The data were collected by a Webropol questionnaire that included both structured and open-ended questions. The questionnaire included background questions about the respondents’ age, medical education (physician or dentist), work experience after obtaining their basic academic degree, the current stage of specialist medical or dental training and their current job description. Open-ended questions were used to clarify the participants’ perception of management and leadership (“What do you think management and leadership means?”) and their thoughts about their management and leadership role in their future career (“What kind of career as a specialist physician/specialist dentist do you dream of, and how do you think your career will develop? What role will management play in your future career path?”). In the final part of the study, the respondents were asked to describe their perceptions of how the roles of an expert/clinician in their specialty and the leadership of a multiprofessional team are emphasized in the work of medical or dental specialists. They were asked to separately reflect on their current experiences and their expectations for the future, looking ten years ahead.

The link to the questionnaire was available on the website of the course. The training participants received a brief introduction about this study during their first online meeting. The link was open for seven weeks from September 12 to October 27, 2023. The study participants were reminded of the study twice. The target group was informed that participation was completely voluntary.

The mean age of the participants in the study group was 33.6 years (SD 4.81), with a median age of 34.0 years and an age range of 27–44 years (eight responses missing). Most of the respondents were physicians (*n* = 29, 78.4%). The number of dentist respondents was eight (21.6%). The average work experience after completing basic education as a physician or dentist was 6.0 years (SD 3.86, range 1.5–18 years), with four responses missing. More than half of the respondents had four and a half years or less of work experience. All respondents were employed in specializing duties at hospitals or public health centers. The current level of completion of their specialist medical or dental training (percentage of studies completed) varied significantly, with a mean of 46.9%, a median of 40% and a range of 10–100% (two responses were missing).

### Analysis

For quantitative analysis (sums, percentages, means, standard deviations), IBM SPSS Statistics version 29.0.2.0 was used. In this study, physician and dentist respondents were analyzed as a single group. The data derived from the responses to the open-ended questions were analyzed using quantitative content analysis as follows. Initially, each data set was read through multiple times. The respondents’ expressions, namely, words, phrases and sentences, served as the units of observation.

The first open-ended responses about the participants’ perceptions of the management were categorized according to Mintzberg’s Managers’ Working Roles model (Mintzberg, 1980, pp. 56–91). Mintzberg classified the major dimensions of managerial work into three categories:

1. interpersonal roles (figurehead, leader, liaison);
2. informational roles (monitor, disseminator, spokesperson); and
3. decisional roles (entrepreneur, disturbance handler, resource allocator, negotiator).

Mintzberg’s model of ten managerial roles offers a comprehensive framework for analyzing what managers actually do (Table 1).

**Table 1.** Participants’ perceptions of managerial work according to Mintzberg’s Manager’s Working Roles model

Main categories	Working roles	*n*	Examples of open-ended responses (case number of respondents)
Interpersonal roles	FIGUREHEAD	1	“For example, being in a position at the workplace where one has authority over others” (22)
	Symbolic head; obligated to perform a number of routine duties of a legal or social nature		
	LEADER	33	“Guidance of people and work that helps the group perform better than without guidance and when working alone” (3)
	Responsible for the motivation and activation of subordinates, responsible for staffing, training and associated duties		
			“Leadership is an activity aimed at achieving results through and with people, focusing on very people-centered work” (13)
	LIAISON PERSON	1	“In my opinion, leadership means understanding the managed unit as a whole, the interactions within it, as well as its interaction with the environment” (14)
	Maintains self-developed network of outside contacts and informers who provide favors and information		
Informational roles	MONITOR	3	“Leadership makes use of the right information about the population using services, the personnel, and the available resources” (4) “… includes awareness of goals and resources” (14)
	Seeks and receives wide variety of special information (much of it current) to develop thorough understanding of organization and environment; emerges as nerve center of internal and external information of the organization		
	DISSEMINATOR	1	“Effective communication and information sharing” (18)
	Transmits information received from outsiders or from other subordinates to members of the organization; some information factual, some involving interpretation and integration of diverse value positions of organizational influencers		
	SPOKESMAN	3	“Outwardly, [the leader] represents the team’s activities, while at the same time advocating for their team” (8)
	Transmits information to outsiders on organization´s plans, policies, actions, results, etc.; serves as expert on organization´s industry		
			“The leader serves as a link between the employees and the upper management” (20)
Decisional roles	ENTREPRENEUR	7	“Development of the work unit’s operations and planning of reforms as needed” (2)
	Searches organization and its environment for opportunities and initiatives “improvement projects” to bring about change; supervises design of certain projects as well		
			“Development work is a major part of leadership” (20)
	DISTURBANCE HANDLER	4	“Solving problematic situations” (2)
	Responsible for corrective action when organization faces important, unexpected disturbances		“The work includes guiding the unit being led towards goals, resolving conflicts, and considering the big picture” (14)
	RESOURCE ALLOCATOR	24	“The leader takes into account the skills and strengths of subordinates and utilizes them when developing work” (3)
	Responsible for allocation of organizational resources of all kinds - in effect the making or approval of all significant organizational decisions		
			“Leadership is the organization of things and making decisions” (17)
	NEGOTIATOR	0	
	Responsible for representing the organization at major negotiations		

**Source(s):** Original descriptions from Mintzberg (1980), pp. 56–91; Authors’ own work

The second set of open-ended responses of the respondents’ thoughts on their future career development and the role of management in their future career were categorized into four categories: Yes (clear interest in management), Maybe (could possibly be part of their job description), in any case (considering that management will be part of one’s future job description, even if management was not deemed interesting in itself) and No (clear lack of interest in management or interest in role focused exclusively on clinical practice) (Table 2).

**Table 2.** Respondents’ descriptions of their interest in management and leadership roles in their future career

Interest in management	*n* (%)	Examples of open answers (case number of respondents)
Yes	8 (21.6)	I would like to be the leader/chief dentist of a small organization, who also performs clinical work” (6)
		“I would be able to utilize my professional skills in tasks that interest me. I think that, at some point, I want to move into some kind of a leadership role” (21)
		“I envision a career more focused on clinical work at this stage, but as my skills grow, the job will typically involve more leadership elements, for example, guiding the nursing staff and less experienced colleagues” (22).
		“I dream of a long progressive career, and management work will have a role in that” (23)
Maybe	10 (27.0)	“What’s essential for me is to develop into a good specialist. If necessary, if time allows, I could also consider a chief physician role” (9)
		“At this stage, I consider specialization important for the development of my professional skills, so I want to start performing and developing clinical work in my specialty as comprehensively as possible. However, leadership work is not out of question. To some extent, it’s likely that I will have to do some leadership work in the future” (26)
		“Because I want to operate in various locations, I do not see formal leadership work as something I identify with, but in individual forums, I can see that I could lead some so-called working groups” (27)
In any case	9 (24.3)	“I suspect that the narrower the area one aims to become a top expert in, the stronger their position will be as a leader in the care of these patients within a multidisciplinary team and in taking responsibility for the operations of the units” (2)
		“Primarily clinical work, but in my specialty, the work is team-based, so serving as a team leader comes naturally with it” (12)
		“I would like to work as an expert in clinical work and hope that the role of leadership will be as small as possible” (19)
No	10 (27.0)	“I do not dream of leadership; I would like to work as a specialist dentist” (1)
		“I dream of a career where I am someday an expert and a specialist physician. I am progressing towards my goals by completing my specialization. I am not currently interested in a managerial position” (7)
		“I could be interested in development work later, but at least for now, I do not see myself in a managerial role” (20)
		“Purely from the perspective of a clinician” (37)

**Figure 1.**
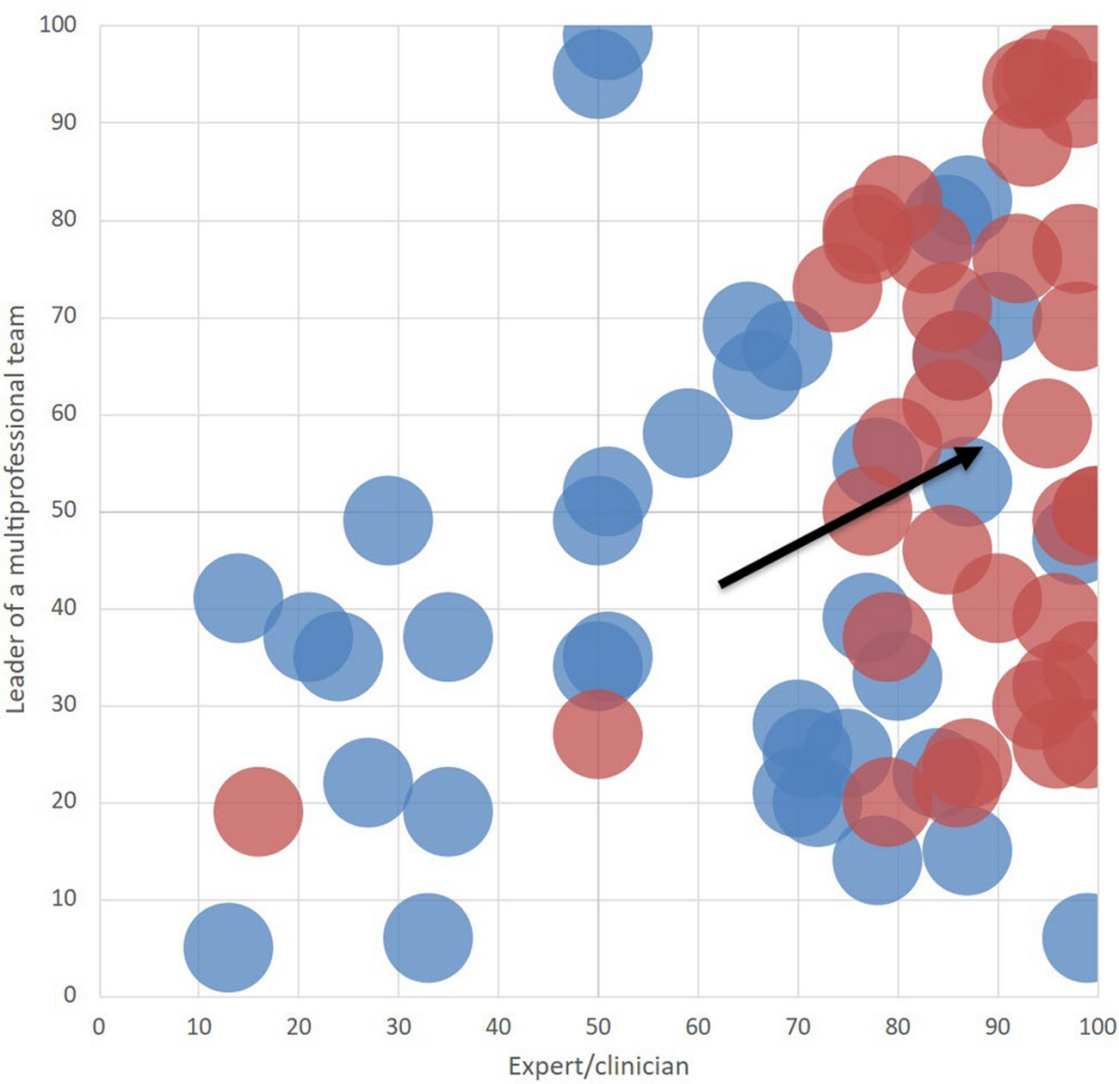
Scatter plot illustrating respondents’ perceptions of how they emphasize their roles as experts/clinicians within their specialty and as leaders of a multiprofessional team. Each respondent’s current situation is represented by a blue bubble, and their desired situation in 10 years by a red bubble. The average change across all respondents is indicated by an arrow **Source:** Authors’ own work

**Figure 2.**
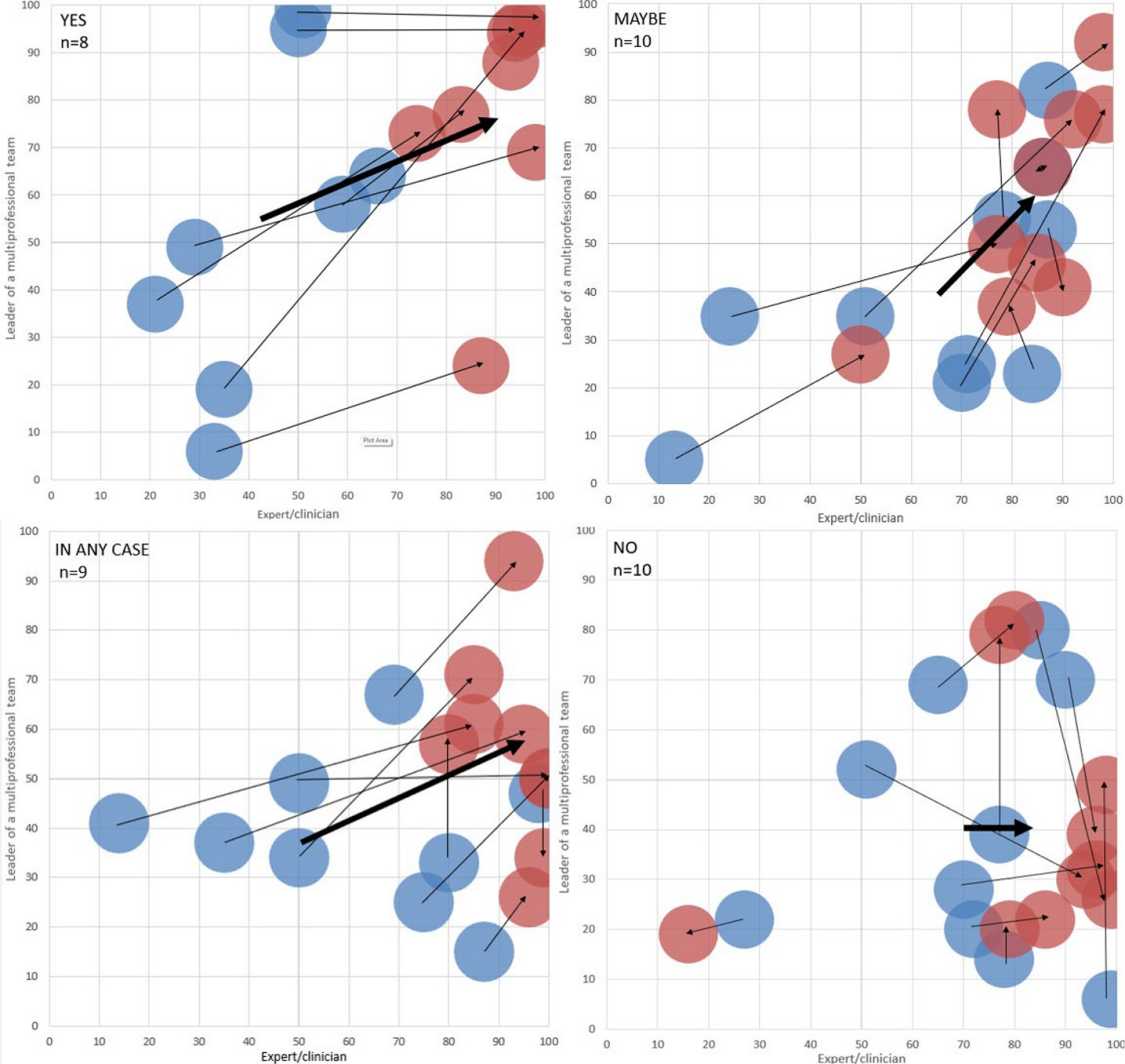
A scatter plot illustrating the four different manager/leader role options considered by respondents in their career reflections. The respondents’ initial situation (blue bubble) and the desired situation in the future (10 years) (red bubble) and individual changes and the average of all respondents are depicted by arrows **Source:** Authors’ own work

A scatter plot was used to describe participants’ perceptions of how the work of a specialist/clinician in their specialty and the leadership of a multiprofessional team are emphasized in the work of a medical specialist, both in the current situation and ten years from now. The *y*-axis represented the role of the team leader, while the *x*-axis represented the role of the expert. These were first analyzed for the entire study group (Figure 1) and, subsequently, the perceptions were categorized into four groups based on the participants’ estimations of their future management role (Yes, Maybe, In any case, No) (Figure 2).

### Ethics

The study was conducted in accordance with good scientific practice and research ethics. Participation in the study was voluntary, and the research design did not require prior ethical review (The Finnish National Board on Research Integrity, 2019). This is because, in Finland, the support of the Ethics Committee is not required for research in which data is collected using a voluntary questionnaire. The study participants were informed of its purpose, and the data collected in the study were not used for other purposes. The questionnaire included a data privacy statement. The participants provided informed consent in the questionnaire, allowing their responses to be used for research purposes. The results have been analyzed and reported in a manner that ensures that no individuals can be identified, even based on background information. All collected data and research findings have been processed and stored confidentially, in accordance with the ethical guidelines of the University of Eastern Finland.

## Results

### Perceptions of management and leadership

Among Mintzberg’s managerial roles, the leader role was the most frequently present in the participants’ responses (*n* = 33). Participants described the work of leaders as guiding and organizing personnel to achieve improved outcomes. The second most frequently identified expressions were associated with the resource allocator role (*n* = 24). Expressions related to the resource allocator role involved considering the skills and strengths of subordinates to support work development, organizational management and decision-making. The entrepreneur role was identified less frequently (*n* = 7) and included descriptions of managers who initiate and plan reforms. Expressions of the other roles in the interpersonal and decisional categories were found considerably less frequently. Finally, only a few expressions representing the informational roles category were found (Table 1).

### Interest in the management and leadership role in the future

The participants had varying expectations regarding their careers and future roles in management and leadership. Three-quarters of the respondents had a positive outlook on management and leadership. Some expressed a clear interest in pursuing management and leadership roles but indicated that they preferred to focus on their clinical careers first. Some of the participants expressed a stronger interest in clinical work, but also considered it possible for them to assume a management role, at least as a team leader (Maybe). Some participants had realized that management would be part of their future work, even though they did not find management particularly interesting in itself (In any case). Finally, about one-fourth of the participants had a clear lack of interest in management and leadership, or were purely interested in a clinician role (No) (Table 2). Compared to the other three groups combined (those with a positive outlook on management and leadership), the respondents in the No group were, on average, older (35.8 years versus 32.7 years), more experienced (7.9 years versus 5.5 years) and had made further progress in their specialist training (55.0% versus 43.6%).

### Perceptions of expert and team leader roles

At the whole group level, there was a substantial difference between the current situation and future expectations, as the respondents anticipated a significant shift toward stronger expertise (from 61.3% to 86.7%) and increased team leadership roles (from 42.6% to 56.9%) (Figure 1).

While divided by the respondents’ perceptions of the management and leadership role in their future careers, there was a stronger increase in expertise than in team leadership in all groups, except for the Maybe group, where the increase was almost equal. The Yes group had the lowest mean starting point for the expert role. The team leadership role increased in all groups except for the No group. The average change was the most substantial in the Yes and in the In any case groups, and the least substantial in the No group. However, there were considerable individual variations within the groups, as shown in Figure 2.

## Discussion

This study aimed to examine specializing physicians and dentists’ perceptions of management and leadership, their interest in managerial work and the role of management in their future careers.

Respondents’ views on management roles included perceptions of the roles of leaders, resource allocators and entrepreneurs. One-quarter of the respondents were clearly interested in management and leadership, and, overall, three-quarters of the respondents believed that management and leadership would play a role in their future careers. As could be expected, the respondents’ thoughts and assumptions about their future roles as specialized physicians or dentists suggest that, ten years from now, the role of expertise will become significantly stronger. However, they also anticipated that their management and leadership responsibilities would become more prominent.

### Perceptions of management and leadership: focus on leading people

The study respondents were asked to reflect on what leadership and management involve. Their responses were classified according to Mintzberg’s Manager’s Working Roles model (Mintzberg, 1980, pp. 56–91). This structure clarifies the complexity of managerial work beyond job titles, illustrating how managers balance diverse demands and affect organizational functions. Mintzberg’s model is applicable across industries, serving as a credible foundation in studies of real-world management and supporting evaluations of performance in areas such as leadership, communication, decision-making and resource allocation.

The respondents’ characterizations were found to primarily fall into two categories: interpersonal and decisional roles. Of the interpersonal roles, the leader’s role was the most frequently identified. Similar results were found in another study of specializing physicians and dentists (Kuivalainen *et al.*, 2024). Surprisingly, little attention was paid to the role of a liaison person, despite considering networking as an important resource for managers and leaders (Tuononen *et al.*, 2023). It is likely that the respondents, still in specialist training and with limited or no management experience, have not yet fully recognized the importance of networking in managerial roles.

Of the decisional roles, the resource allocator role emerged. While this is not surprising, one might wonder to what extent the scarcity of healthcare funding and other resources, especially professionals, along with current funding cuts in Finland, have shifted attention toward a focus on what is missing, those resources that cause concern. One in five respondents made reference to the entrepreneur role. This role may be undervalued, as development work could be perceived as an important aspect of management. Surprisingly, there were only a few mentions of informational roles, despite a shared experience of gaps in the flow of information. By contrast, Kuivalainen *et al.* (2024) found that the disseminator role was perceived as an important informational role in a similar study group.

### Perceptions of the management and leadership role in the future: positive outlook on management and leadership

Four distinct perceptions emerged that describe the respondents’ interest in the role of management and leadership in their future careers. Almost three out of four respondents foresee management, at least to some extent, as a part of their future careers. The extremes were the Yes group, demonstrating a clear interest in management, and the No group, which displayed a distinct lack of interest in management or focused solely on a clinician’s role. Approximately half of the respondents believed that it might be possible for them to assume a management position, at least as a team leader (the Maybe group), or that management could be part of their future job description, even if they did not find management interesting in itself (the In any case group). Thus, the outcome may be the same for these two groups, but their approaches differ: the Maybe group perceives a managerial role more as an opportunity, whereas the In any case group regards it more as an obligation.

A comparable result was found in a study conducted among respondents in a specialist training program similar to that in the present study. Around one-fifth of the respondents were clearly interested in becoming leading physicians, whereas a similar proportion indicated that they might be interested. Around half of the respondents expressed no interest, but some qualified their responses by adding that they were “not interested right now” or “not at the moment” (Kuivalainen *et al.*, 2024). Most of the more experienced and already specialized physicians were found to be interested in management roles in their future careers, according to Parviainen *et al.* (2024). Huikko-Tarvainen *et al.* (2021) studied final-year medical students, and a little over half of their participants were either interested or somewhat interested in becoming physician leaders, while just a little under half were not interested. It is clear that doctors at different stages of their careers mainly recognize management tasks as part of their job and are also interested in them.

According to responses from all groups, the primary desire that emerged from the data involved working as a clinician. The respondents did not have a lot of work experience yet, and their strongest work experience had been gained in an expert role. They had been trained as experts in professional fields such as medicine and dentistry and were currently further specializing in their field and advancing their expertise. At the beginning of their specialist careers, these individuals are likely to still need to gather more experience to clearly understand what they truly want from their professional paths (Schein and van Maanen, 2016; Schein, 1990).

While many respondents probably lacked personal experience in managerial roles, all had experience of being managed by others. Although many of the respondents did not find management particularly interesting, they still acknowledged that it would be a part of their future work. Based on these results, three-quarters of the respondents currently viewed leadership as a component of their career outlook. However, it remains to be seen how their careers will actually develop in the future. Overall, they were still highly unfamiliar with what working in a specialized field entails.

Previous studies have shown that physicians and dentists enter managerial positions for various reasons. Some are motivated by a personal desire, while others may end up as managers by chance, force or circumstance (e.g. a distinguished clinician may be assigned the role) (Ham *et al.*, 2011; Savage *et al.*, 2020; Tuononen, 2018, pp. 27–28). In addition, this may be the only vertical career advancement model within hospital organizations. Can we assess the future interest in managerial positions based on these results? For example, can we expect higher-quality managers to emerge from the “Yes” group? What about those unwilling to take on or with no interest in leadership roles, but who end up in those positions anyway? The “No” group consisted of, on average, those who were older, more experienced and further along in their specialization training. Could this reflect cynicism related to the work experience already gathered and a reluctance toward leadership work? (compare Tuononen, 2018, pp. 28–29).

It is assessed that the basic characteristics of physicians would render them good healthcare leaders (Angood *et al.*, 2014; Sarto and Veronesi, 2016). It is important that leaders are not only competent but also interested in management and leadership. Completing specific training in management and leadership is found to be crucial (Parviainen *et al.*, 2018; Satiani *et al.*, 2014; Silbaugh and Leider, 2009). Appointing top clinicians to leadership positions carries the risk of poor outcomes if they are unwilling or unprepared to lead. In such cases, organizations not only fail to gain effective leaders but may also lose highly skilled clinicians.

### Perceptions of expert and team leader roles: high expertise, moderate leadership

In this study, there was variation in the respondents’ views on changes in their careers related to expertise and team leadership. On average, there was a clear expectation of increased expertise and leadership roles, with most respondents experiencing growth in these areas. Across all groups, the primary desire was to continue working as clinicians. This was to be expected, as our respondents were still participating in their specialty training, and management and leadership training integrated into their studies was still ongoing. In addition, the respondents were not yet fully aware of their future job descriptions. For physicians and dentists, balancing managerial work with clinical duties is often challenging (Crocitto *et al.*, 2021; Huikko-Tarvainen *et al.*, 2019; Huikko-Tarvainen *et al.*, 2021; Spehar *et al.*, 2015; Tuomiranta, 2002, p. 121; Tuononen, 2018, p. 37; Virtanen, 2010; Vistbacka, 2019, p. 65). Many physicians in leadership positions continue clinical work to enhance their credibility and maintain a connection to clinical practice, which is often considered less hectic than leadership responsibilities (Ham *et al.*, 2011; Huikko-Tarvainen *et al.*, 2019). Due to a strong physician identity, physicians often perceive themselves as leaders only secondarily (Andersson, 2015; Lönnqvist, 2014; Sullivan *et al.*, 2022). The dilemma of balancing expert and leadership roles is widely recognized, and a similar phenomenon has been observed in other expert professions (Heilmann, 2004).

### Strengths and limitations

The topic of this study is very timely. Management and leadership are at the forefront in healthcare, especially at this moment, when there are major organizational changes underway. The specific challenges include various needs for reform, as well as issues related to resources and finances. The respondents will be specially trained future healthcare experts and leaders, which makes their perspectives relevant and provides valuable insights into the topic of the study.

The response rate (32.5%) can be considered reasonable for an online survey. The wording of the second open-ended question guided the respondents to discuss their career dreams and career development plans from a leadership perspective. Although the focus of this study was on management and leadership, a different framing of the question might have revealed other career perspectives in the responses.

## Conclusion

The healthcare sector needs physician leaders. It is therefore desirable that physicians show an interest in leadership. Although the participants in this study had not yet completed their specialization training, the majority of them already believed that leadership could be part of their future work as specialists and dentists. Although expertise will be emphasized in their careers, leadership and supervisory roles will also become increasingly important. Those interested in leadership work need support to allow them to develop their leadership skills without the experience of a conflict between clinical work and management work. This topic is worth studying in the future as well − perhaps among specialized participants to gain more insights into working as a medical or dental specialist and the role of leadership in that work.
